# Unilateral biportal endoscopy via two different approaches for upper lumbar disc herniation: a technical note

**DOI:** 10.1186/s12891-024-07339-8

**Published:** 2024-05-10

**Authors:** Rongxue Shao, Weibin Du, Wei Zhang, Wei Cheng, Chengyue Zhu, Jiaming Liang, Jun Yue, Hao Pan

**Affiliations:** 1 Department of Orthopaedics, Hangzhou Traditional Chinese Medical Hospital, Zhejiang Chinese Medical University, Hangzhou, 310007 Zhejiang China; 2 Research Institute of Orthopedics, the Affiliated Jiangnan Hospital of Zhejiang Chinese Medical University, Hangzhou Xiaoshan Hospital of Traditional Chinese Medicine, Hangzhou, 311201 Zhejiang China

**Keywords:** Percutaneous endoscopic, Interbody fusion, Lumbar disc herniation, Surgical approach, Unilateral biportal endoscopy

## Abstract

**Background:**

The traditional surgical procedures for upper lumbar disc herniation (ULDH) usually lead to frequent complications. We aim to investigate the clinical efficacy of the unilateral biportal endoscopy (UBE) technique in treating upper lumbar disc herniation (ULDH).

**Methods:**

From January 2020 to December 2021, the clinical data of 28 patients with ULDH treated with the UBE technique were collected and analyzed for surgery time under UBE, postsurgical drainage, postsurgical hospital stay, and complications. The clinical efficacy was evaluated according to the modified MacNab score, Oswestry disability index (ODI), and visual analogue scale (VAS) of low back pain and lower limb pain before the surgery; one week, one month, and three months after the surgery; and at the last follow-up.

**Results:**

All patients underwent the UBE surgery successfully. The surgery time under UBE for non-fusion cases was 47.50 ± 11.84 min (monosegment) and 75.00 ± 20.66 min (two segments), while that for fusion cases was 77.50 ± 21.02 min. The postsurgical drainage for non-fusion cases was 25.00 ± 13.94 mL (monosegment) and 38.00 ± 11.83 mL (two segments), while that for fusion cases was 71.25 ± 31.72 mL. The postsurgical hospital stay was 8.28 ± 4.22 days. The follow-up time was 15.82 ± 4.54 months. The VAS score for each time period after the surgery was significantly lower (*P* < 0.05), while the ODI was significantly higher than that before the surgery (*P* < 0.05). According to the modified MacNab scoring standard, the ratio of excellent to good was 96.43% at the last follow-up. Two patients experienced transient numbness and pain in their lower limbs and no activity disorder after the surgery, and they recovered after conservative treatment.

**Conclusions:**

The clinical effect of UBE technique in treating ULDH was reliable. According to the needs of the disease, the interlaminar approach or paraspinal approach of the UBE technique was selected. This technique took into account the effect of treatment, achieved the purpose of minimal invasiveness, and did not require special instruments. Therefore, it has the potential for clinical application.

## Introduction

The definition of upper lumbar disc herniation (ULDH) is controversial. Some authors have reported upper lumbar discs as T12–L1, L1–L2, L2–L3, and L3–L4 [1], while some other studies have considered them as only L1–L2 and L2–L3 [2]. The spinal canals of upper lumbar discs are narrower than those of the lower ones, which may compromise multiple spinal nerve roots or conus medullaris and lead to complex and variable signs [3]. The traditional treatments for ULDH include open posterior lumbar interbody fusion [4], transforaminal lumbar interbody fusion [5], and posterior decompression [6], and only nucleus pulposus enucleation via intervertebral foramen (or fenestration of laminar space). The aforementioned open surgery had the advantages of clear vision and reliable efficacy. However, surgical procedures usually lead to frequent complications, such as larger iatrogenic trauma, higher bleeding volumes, and a longer hospitalization time because of the need for extensive dissection of the surrounding muscles, fascia, and ligaments. Additionally, they often affect the daily life and work of the patient because of complications such as persistent back pain, spinal stiffness, and weakness [7].

The development of endoscopic technology and equipment has enabled wide acceptance of endoscopic treatment by spinal surgeons and patients because of its advantages of smaller iatrogenic trauma, less blood loss, shorter hospitalization time, and satisfactory clinical effect [8]. At present, the endoscopic treatment for ULDH mainly includes microendoscopic discectomy and percutaneous transforaminal endoscopic discectomy. In both cases, discectomy and intervertebral fusion are performed through a single portal. They have the advantages of smaller iatrogenic trauma, less bleeding, and rapid recovery [9]. However, both of them are single-portal surgery, that is, the view portal and the work portal are in the same frame and coaxial. The disadvantages of this endoscopic technique include poor stereoscopic positioning, limited range of movement of instruments, and low work efficiency. At the same time, special surgical instruments and equipment are required, limiting its further development.

Therefore, some scholars proposed the application of biportal endoscopy with intraoperative irrigation in treating lumbar disc herniation and lumbar spinal stenosis and achieved good results [10]. The two portals used in this method are the view portal and the work portal. The endoscope is placed in the view portal to obtain a clear surgical field of vision. Related operating tools, such as the plasma radiofrequency wand, grinding drill, lamina biting forceps, and nerve stripping, are used to complete various surgical operations inside and outside the spinal canal through the work portal [11]. Intraoperative continuous saline irrigation is used to remove bleeding or waste from the surgical procedure. In addition, the water pressure of irrigation inhibits bleeding and ensures clear vision. The two-portal technology realizes multiangle surgery, greatly improving the defects of the coaxial endoscopic technology such as a narrow field of vision and limited operating angle [12]. At the same time, the surgery can be completed with conventional instruments. Therefore, unilateral biportal endoscopy (UBE) technology is now gradually favored by spinal surgeons and even neurosurgeons.

We retrospectively analyzed data from 28 patients with ULDH (L1–L2, L2–L3, and L3–L4) treated with UBE surgery. According to the different intervertebral spaces and types of lumbar disc herniation, different approaches were selected for the UBE surgery and satisfactory results were obtained. The interlaminar approach is used to treat cases of central or paracentral disc herniation. For patients with foraminal or extraforaminal disc herniations, the paraspinal approach is a good choice. By analyzing the aforementioned data, satisfactory clinical results were obtained with both approaches of UBE technique in treating ULDH. Furthermore, this study further discussed the clinical efficacy and advantages of UBE technology in treating ULDH, and summarized the operating points and precautions of different approaches.

## Methods

### Patient population

The study was conducted in accordance with the guidelines of the Declaration of Helsinki and was approved by the ethics committee of Hangzhou Hospital of Traditional Chinese Medicine Affiliated to Zhejiang Chinese Medical University(NO. HZSZYY-20200310M0168), and written informed consent was obtained from each participant. From January 2020 to December 2021, 28 patients (32 segments) with ULDH in Hangzhou Hospital of Traditional Chinese Medicine were treated with UBE surgery. Of the 28 patients, 16 were men and 12 women, aged 20–87 years, with an average age of 63.2 ± 18.1 years. The presurgical data collected included the presence or absence of back and radicular pain, motor and sensory deficit, and reflex changes. The patient characteristics are listed in Table 1.

**Table 1:** Patient characteristics

No. of discs	2 (L1–L2)	7 (L2–L3)	23 (L3–L4)
Sex	16 (Male)	12 (Female)	
Segment	24 (Monosegment)	4 (Two segments)	
Pain	22 (Back)	26 (Legs)	
Nerve traction test (+)	13 (Femoral nerve)	16 (Lasegue’s sign)	
Sensory deficit	1 (Saddle area)	11 (Legs)	
Reflex changes	5 (Knee tendon)	4 (Achilles tendon)	

### Presurgical evaluation

The eligibility criteria were as follows: (a) diagnosis of monosegment (or two segments) ULDH (L1–L2, L2–L3, L3–L4) confirmed by imaging and clinical symptoms, with or without lumbar spinal stenosis; (b) A VAS score for back or leg pain in a standing position of 4 or more, patients with poor efficacy after conservative treatment for 6 weeks, or patients with central lumbar disc protrusion and prolapse of the spinal canal with severe cauda equina symptoms; and (c) patients with ULDH willing to receive UBE technology treatment and could complete the follow-up for half a year or more.

The exclusion criteria were as follows: (a) Patients with three or more segments of ULDH; (b) patients with ULDH also having adolescent scoliosis or degenerative scoliosis (Cobb angle ≥ 10°); (c) patients with ULDH combined with coagulation dysfunction or taking anticoagulant drugs for a long time; and (d) patients with fresh fracture, infection, tumor, severe osteoporosis, and ankylosing spondylitis in the upper and lower vertebral bodies of the responsibility intervertebral space, and patients with other serious medical diseases who cannot tolerate the surgical trauma.

#### Indications

Interlaminar approach: (a) central or paracentral disc herniations (including protrusion, extrusion, sequestration, recurrent and calcified disc herniation); (b) cauda equina syndrome.

Paraspinal approach: (a) foraminal, and extraforaminal disc herniations (including protrusion, extrusion, sequestration, recurrent and calcified disc herniation); (b) far-out syndrome.

Lumbar interbody fusion: (a) Grade 1 or 2 degenerative or isthmic spondylolisthesis; (b) Central or foraminal stenosis with instability; (c) More than two recurrent disc herniation.

### Surgical procedure

All patients were treated with the UBE technique by the same group of surgeons. Twenty patients were treated with a left approach of the spine, while eight were treated with a right approach. Moreover, the interlaminar approach was considered for 22 patients and the paraspinal approach for 6 patients. Further, 24 underwent non-fusion surgery, and 4 underwent fusion surgery (all fusion cases were of monosegment).

### Posture and anesthesia

Under general anesthesia, the patient was positioned prone on a radiolucent spine operating table. The operating table was adjusted so that the operating intervertebral space was perpendicular to the ground. The patient’s blood pressure was lowered to 90–110 mm Hg/50–70 mm Hg (no less than 70% of the patient’s basal blood pressure) with anesthetic drugs to reduce intraoperative bleeding.

### Presurgical fluoroscopy positioning

**Fig. 1:**
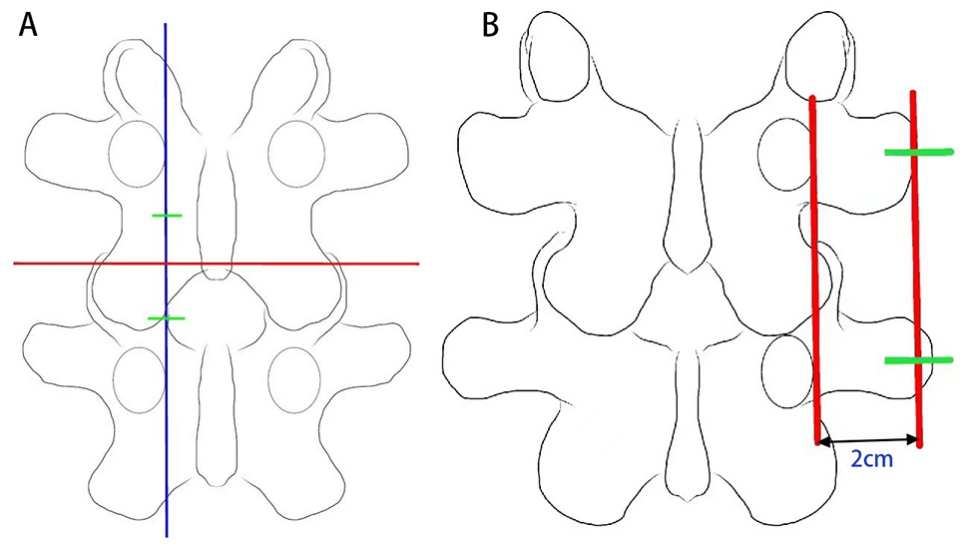
Presurgical positioning of UBE: (**A**) interlaminar approach and (**B**) paraspinal approach

Standard anteroposterior and lateral images of the lumbar spine were obtained under presurgical fluoroscopy. The related intervertebral space was confirmed and focused, and different positioning and markings were selected according to the difference in approach and side. For patients with the interlaminar approach, the initial target point of the endoscope and the instrument was located at the junction of the spinous process and vertebral lamina, and a horizontal line was made around the target point. A marked line was drawn along the inner edge of the pedicle of the upper and lower lumbar vertebrae. Two points, 1.5 cm away from the far and near sides of the junction point of the aforementioned two lines, were used as the body surface positioning points of the view portal and work portal, respectively (Fig. 1A).

For patients treated with the paraspinal approach, a marking line was made along the outer margin of the projection of the pedicle of the upper and lower segments of the responsible intervertebral space. Then, a parallel line was made at a distance of 2 cm from the outer side of the marked line, and a horizontal line was drawn along the isthmus of the superior vertebral body. The two points, located at 1.5 cm on the cranial and caudal sides of the crossing point of the outer parallel line and the horizontal line, were used as the body surface positioning points of the view portal and work portal, respectively (Fig. 1B).

### Surgical procedures

Under the guidance of C-arm fluoroscopy, two transverse skin incisions were made to form the view (cranial side) and work (caudal side) portals. The length of the skin incision was about 1.0-1.5 cm. The surgeon stood on the symptomatic side. A guide rod was inserted into each of the two incisions after the incision of the skin, subcutaneous tissue, and fascia layer. Fluoroscopy determined that the two positioning rods crossed at the ideal target point. Serial dilators were used to separate the back muscle and create the view and work portals. A 30 degree endoscope connected to the irrigation system was inserted through the view portal, and then the irrigation system was turned on for continuous irrigation. The authors preferred to use a natural gravity irrigation system (about 70–100 cm higher above the surgery table) for saline irrigation. The plasma radiofrequency wand was used through the work portal to clean the residual soft tissue on the lamina surface near the target point and control bleeding so as to ensure the clarity of the surgical field.

### Interlaminar approach

The first visual field was the junction between the base of the spinous process and the inferior margin of the lamina of the upper vertebral body. Then, the surgical field was expanded outward and downward by adjusting the direction of the endoscope to fully expose the medial edge of the facet joint and the lamina at the upper and lower edges of the responsible space. Partial hemi-laminectomy was started with an automated drill, and then a laminectomy rongeur or osteotome was used to remove a part of the inferior articular process of the upper vertebral body until the ligament flavum was located cranially and caudally. The ligament flavum adhered to the dural sac was released using a nerve dissector and removed to expose the dural sac. The nerve roots in the corresponding intervertebral space were exposed by sufficient decompression along the surface of the dural sac laterally and caudally. The protruding nucleus pulposus was removed in stages using nucleus pulposus forceps after exploration and separation of the adhesion between the nucleus pulposus and the dural sac. For patients requiring fusion, the tip and medial aspect of the superior articular process (SAP) could be identified and removed. The dural sac and nerve root were pulled to the midline with the assistance of a 1.5-mm-diameter Kirschner wire, and then the residual intervertebral discs and cartilaginous endplate were removed. Autologous bone granules and a cage of appropriate size were implanted into the intervertebral space and fixed with percutaneous pedicle screws. A drainage catheter was routinely inserted to prevent postsurgical epidural hematoma. The skin was sutured layer by layer after extruding the remaining saline for irrigation.

### Paraspinal approach

**Fig. 2:**
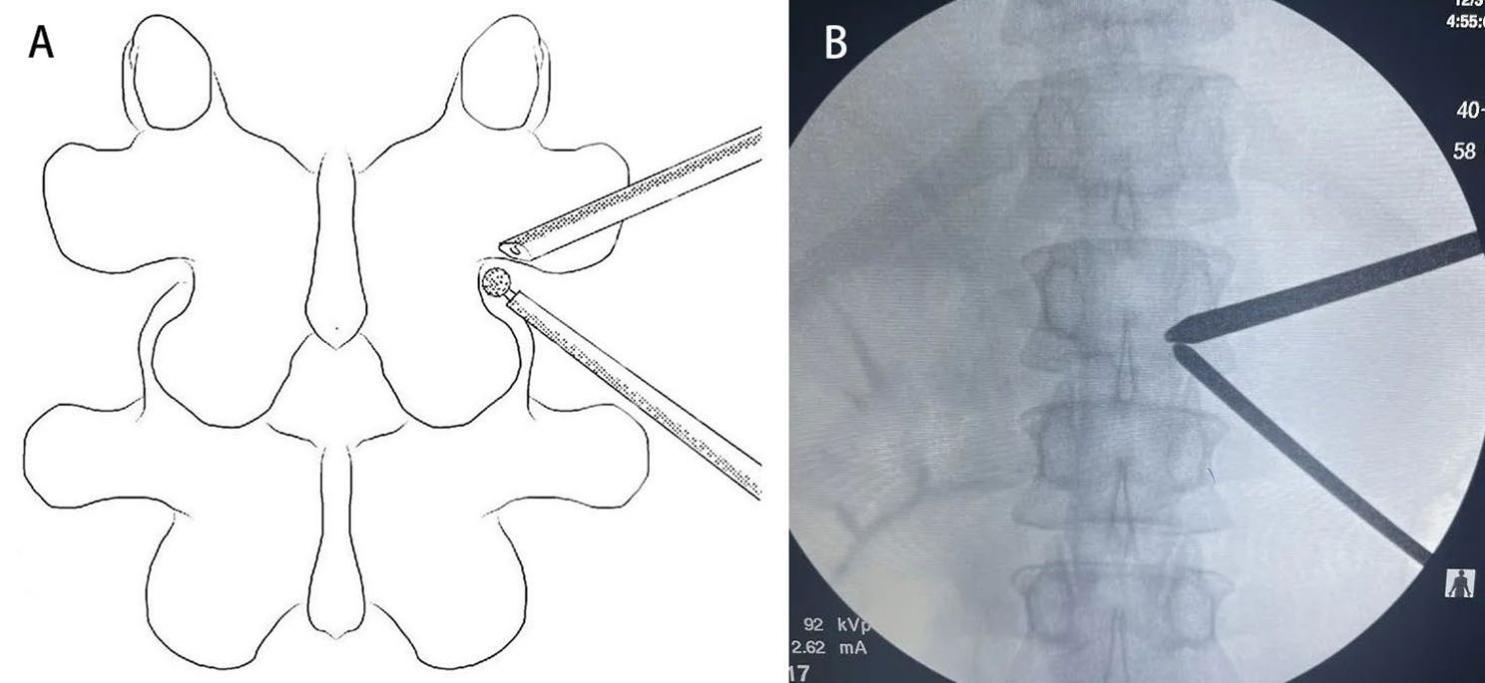
Paraspinal approach: The first visual field was the isthmus

The first visual field was the isthmus. The lateral border of the isthmus was drilled using an automated drill and continued in an under-inside direction (Fig. 2). The tip of the SAP was resected to expose the ligament flavum in the foraminal region. The ligament flavum around the foramen was released using a nerve dissector and removed with a laminectomy rongeur. After completing flavectomy, the exiting nerve root was exposed. The artery around the exiting nerve root was made pre-hemostatic using a radiofrequency wand. The protruding nucleus pulposus was explored using the nerve dissector, and its adhesion with the dural sac or nerve root was stripped. Then, the protruding nucleus pulposus was removed to decompress the exiting nerve root. For the non-fusion cases, the range of articular process joint resection could be controlled according to the size of the drill bit during the surgery. For the fusion cases, the intervertebral space was cleared through the work portal, including the resection of annulus fibrosus, nucleus pulposus, and cartilaginous endplate, and the fusion cage was implanted and placed transversely after intervertebral bone grafting. Then, the pedicle screws were inserted percutaneously. A drainage catheter was finally inserted to prevent postsurgical epidural hematoma.

### Postsurgical management

Low doses of methylprednisolone and prophylactic antibiotic were given intravenously for 3 days after the surgery. The drainage tube was removed when the drainage fluid was less than 50 mL. For the non-fusion cases, the patients were encouraged to walk under the protection of a thoracolumbar brace 24 h after the surgery. For the fusion cases, patients were required to stay in bed for 3 days. The thoracolumbar brace was worn for 1 month for non-fusion cases and 3 months for fusion cases.

The data, including microscopical surgery time, postsurgical drainage volume, postsurgical hospital stay, and complications, were recorded and analyzed. The clinical efficacy was evaluated chiefly using the back and leg pain visual analogue scale (VAS) score, Oswestry disability index (ODI) score, and modified MacNab criteria.

### Statistical analysis

The data were analyzed using SPSS statistical software (version 20.0, SPSS Inc., IL, USA). The multiple logistic regression test was used for the statistical analysis. A repeated-measure univariate analysis of variance was conducted to compare the differences in VAS and ODI scores before the surgery; one week, one month, and three months after the surgery; and at the last follow-up. Statistical significance was set at a probability value of less than 0.05.

## Results

The surgery time under UBE surgery for non-fusion cases was 47.50 ± 11.84 min (monosegment) and 75.00 ± 20.66 min (two segments), and that for fusion cases was 77.50 ± 21.02 min. The amount of postsurgical drainage fluid was 25.00 ± 13.94 mL, 38.00 ± 11.83 mL, and 71.25 ± 31.72 mL, respectively, in case of monosegment non-fusion, two segments non-fusion, and monosegment fusion cases.

### Postsurgical imaging (CT and MRI in non fusion cases, X-ray, CT and MRI in fusion cases)

**Fig. 3:**
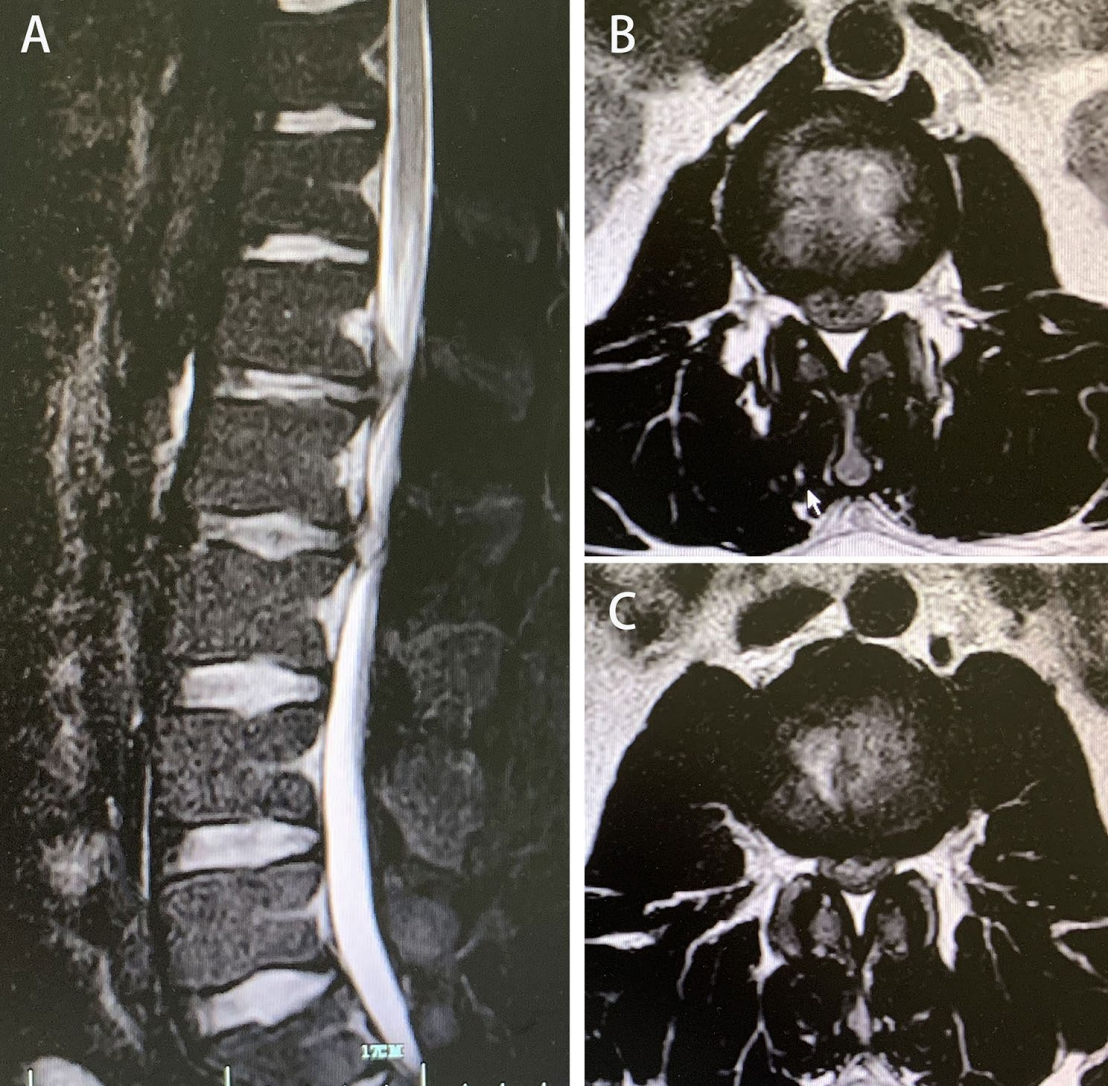
Presurgical magnetic resonance imaging (MRI) showing L1-L2 and L2-L3 disc herniation: (**A**) Sagittal view, (**B**) L1-L2, and (**C**) L2-L3.

**Fig. 4:**
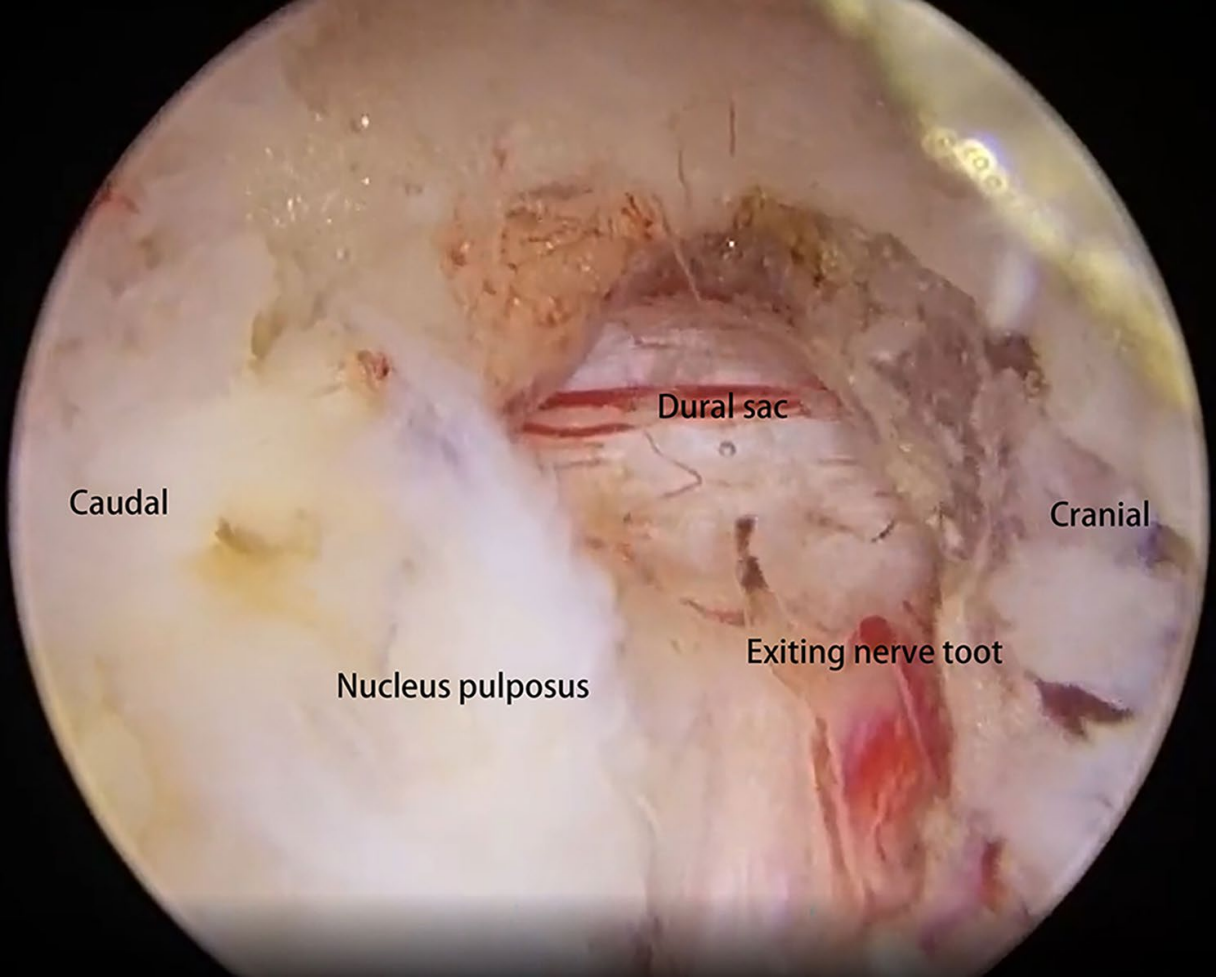
Intraoperative imaging. A nerve dissector was used to explore the breaking of the annulus fibrosus, isolate the adhesions, and expose the herniated nucleus pulposus

**Fig. 5:**
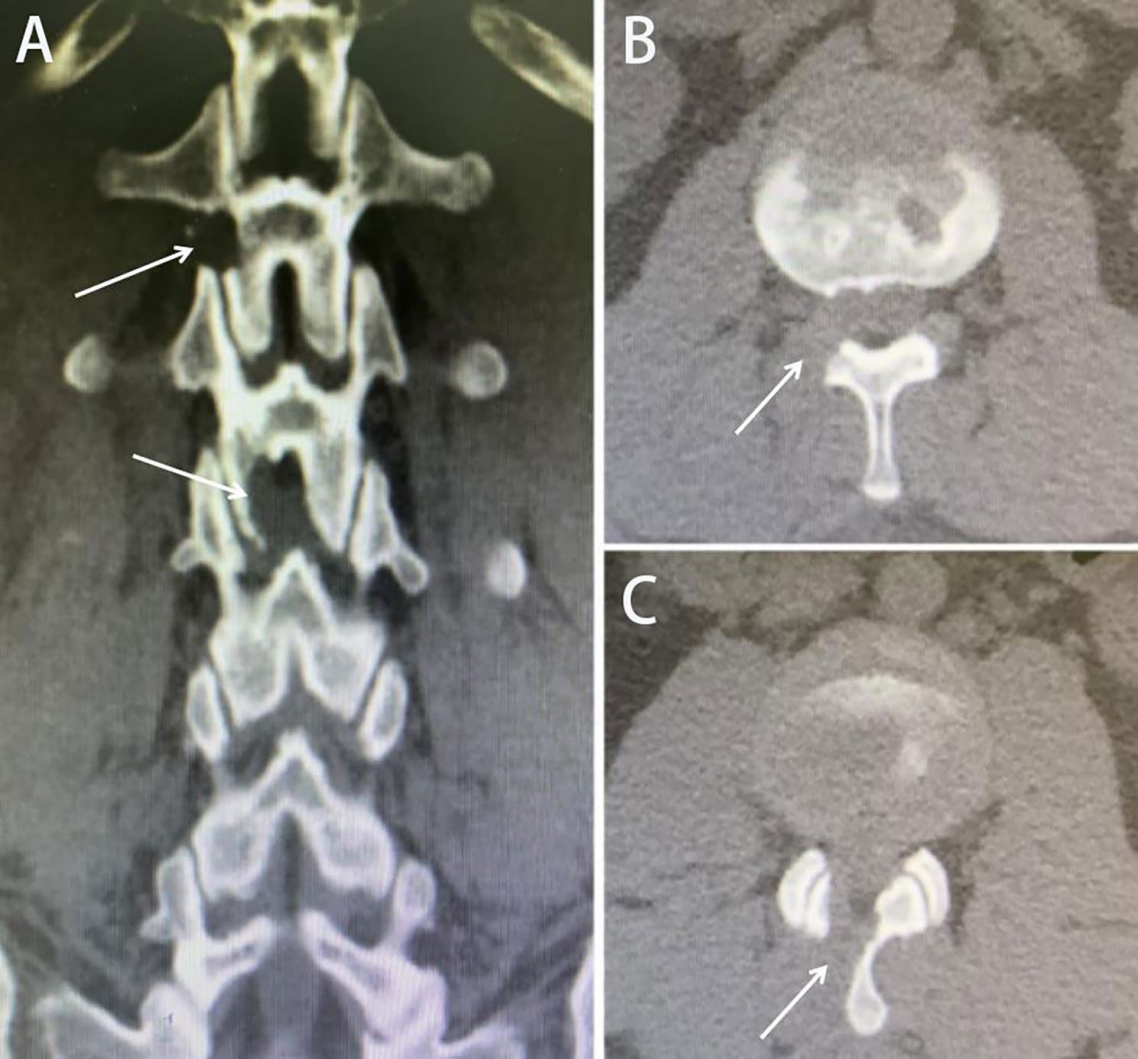
Postsurgical computed tomography (CT) showing the resection range of bone tissue in L1-L2 (paraspinal approach) and L2-L3 (interlaminar approach) (the white arrow). (**A**) Coronal view, (**B**) L1-L2, and (**C**) L2-L3.

**Fig. 6:**
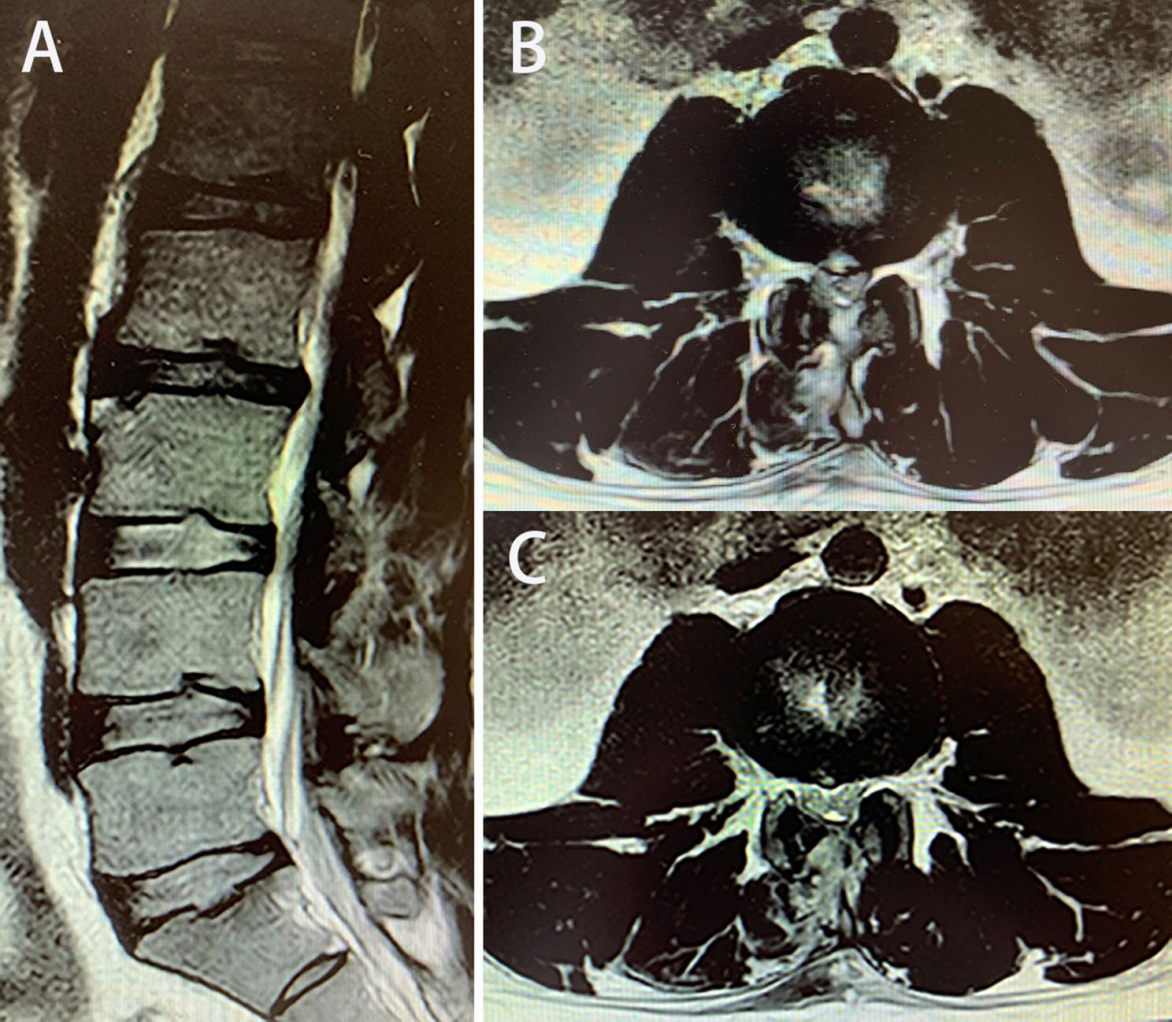
Postsurgical MRI showing that the herniated L1-L2 and L2-L3 intervertebral discs were removed completely and the dural sac was restored to swell. (**A**) Sagittal view. (**B** and **C**) Axial views of L1-L2 and L2-L3.

**Fig. 7:**
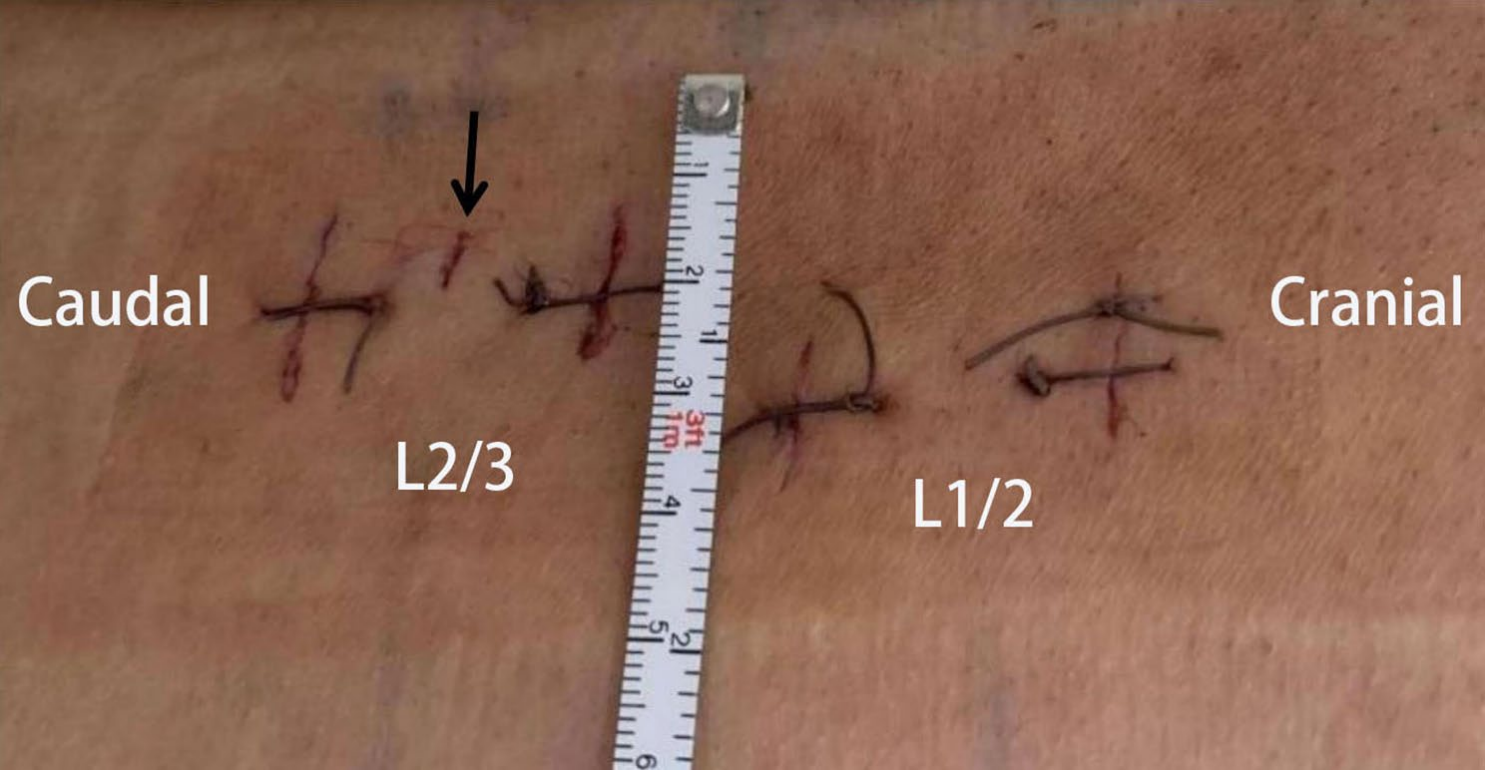
Surgical incisions. The black arrow points to the incision to place the Kirschner wire

**Fig. 8:**
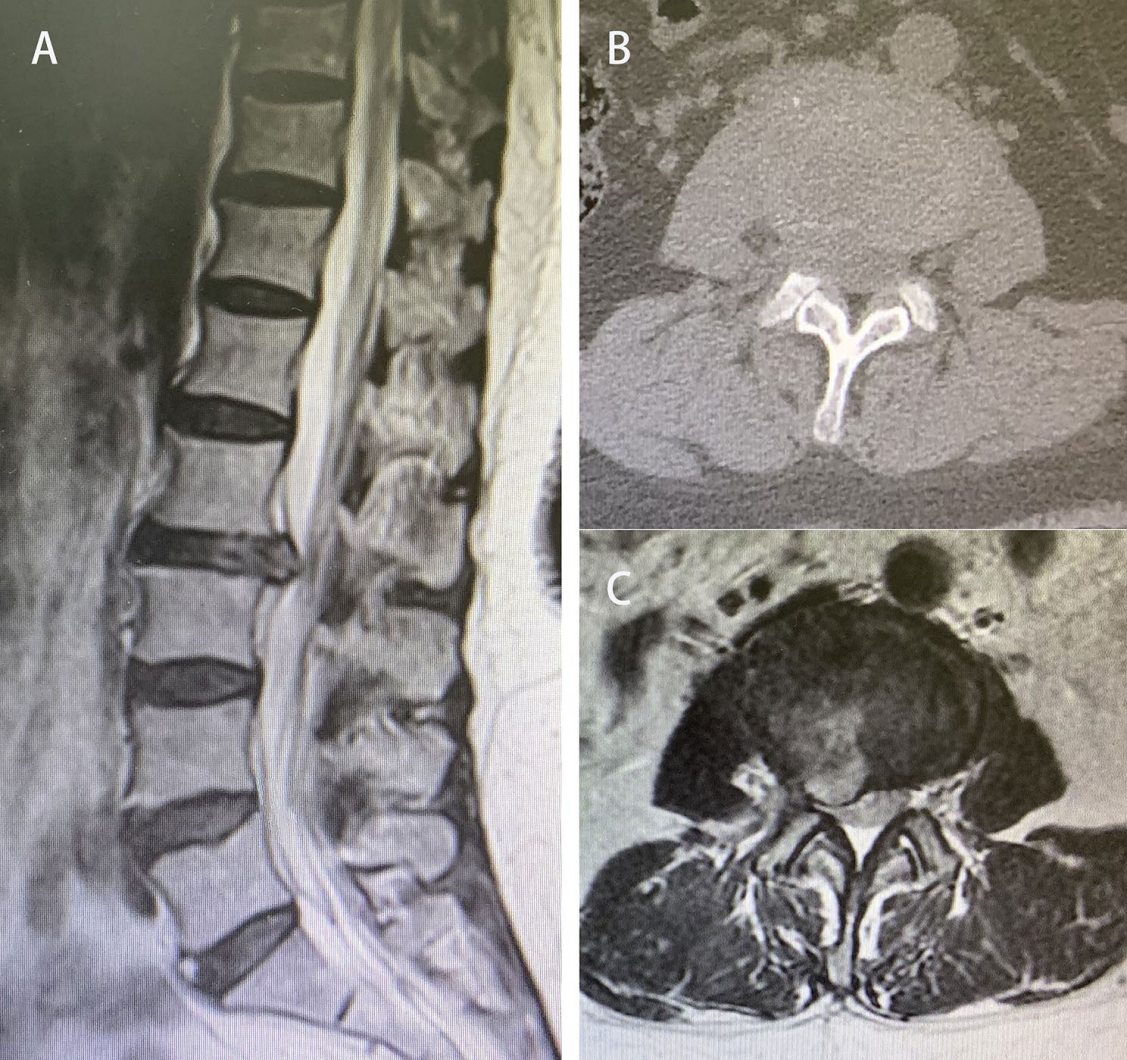
Presurgical MRI showing L2-L3 disc herniation. (**A**) Sagittal view. (**B** and **C**) Axial views of L2-L3 (B, CT; C, MRI).

**Fig. 9:**
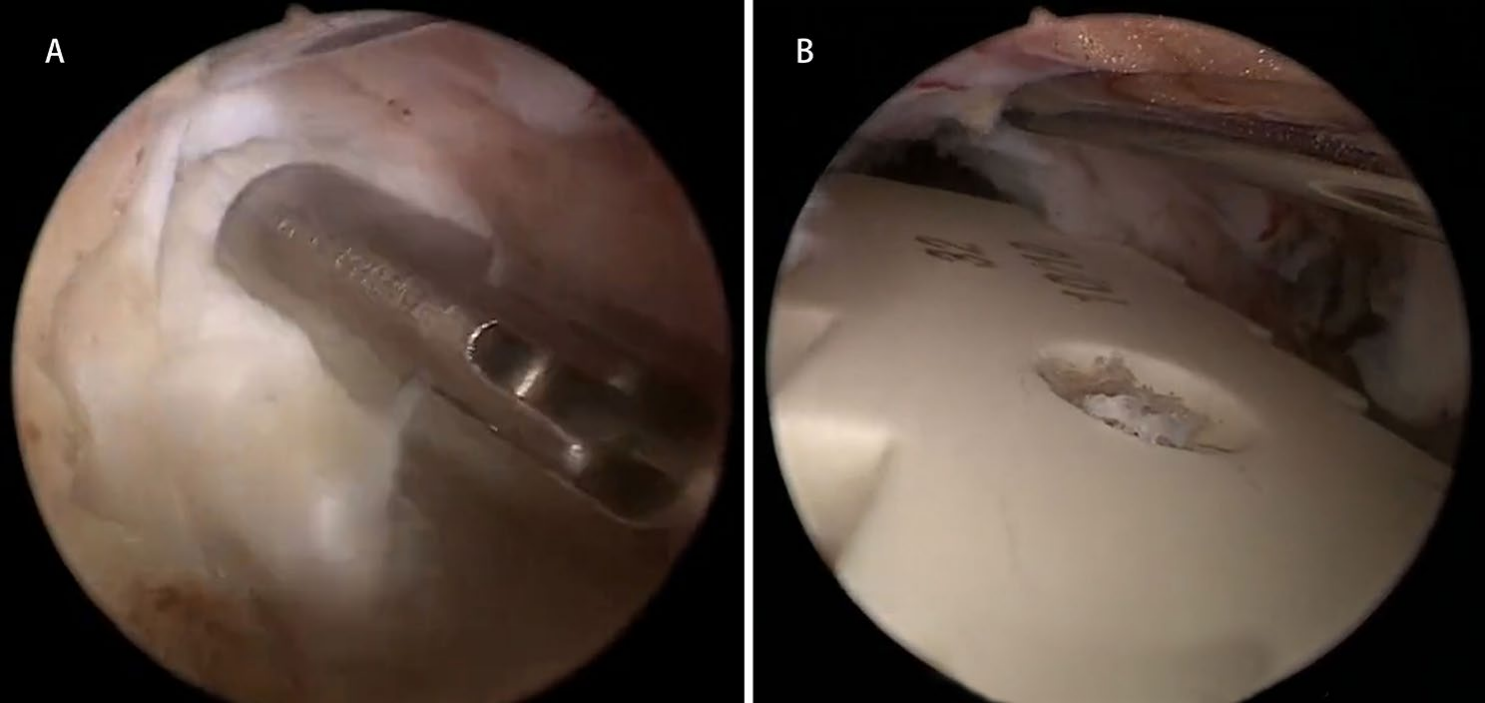
During the surgery, the Kirschner wire was anchored to the upper vertebral body to retract the dural sac, the intervertebral space was cleared, and the fusion device was placed. (**A**) Intervertebral space cleared. (**B**) Fusion device being placed

**Fig. 10:**
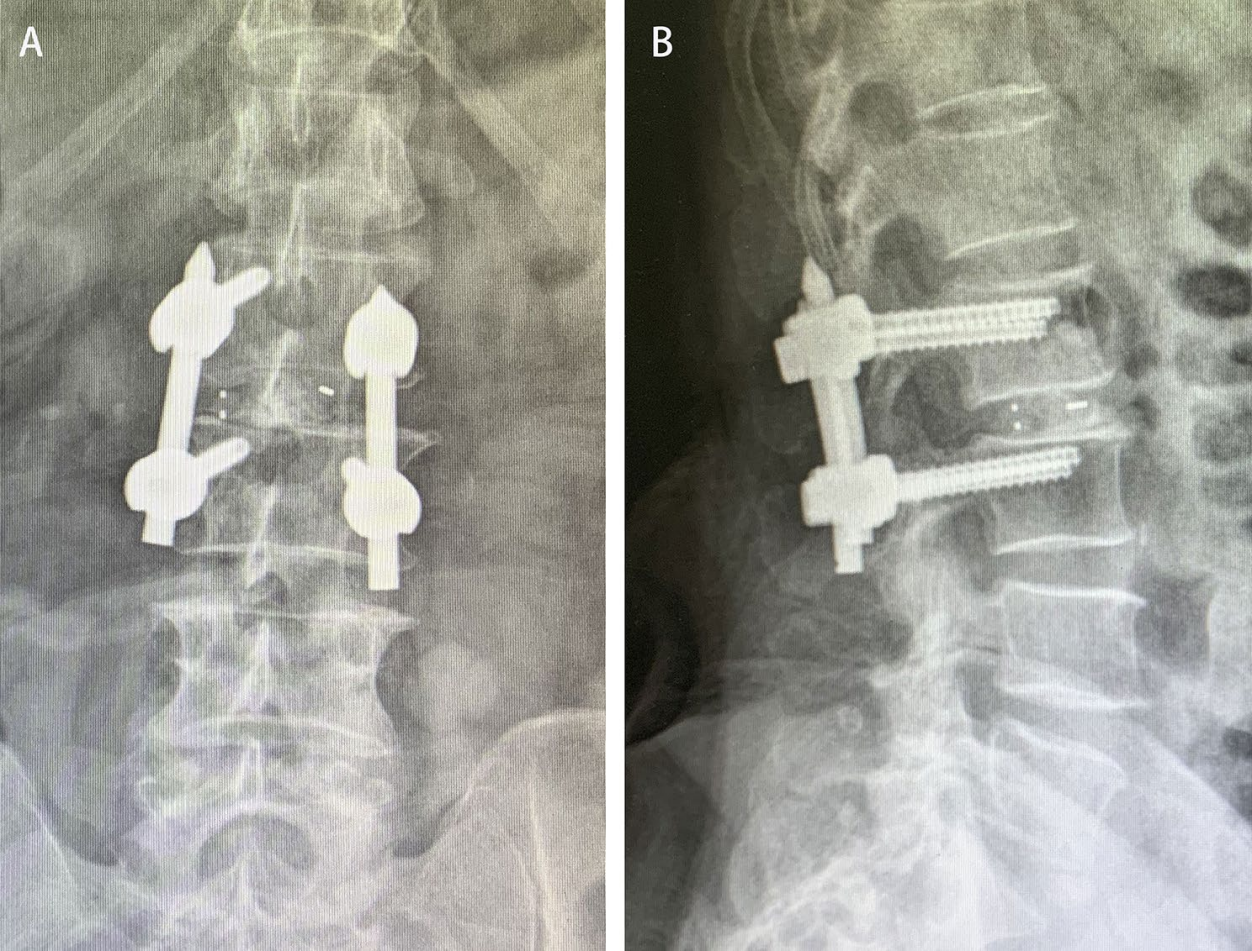
Postsurgical anteroposterior and lateral radiographs showing satisfactory positions of the fusion cage and internal fixators. (**A**) Anteroposterior image. (**B**) Lateral image

**Fig. 11:**
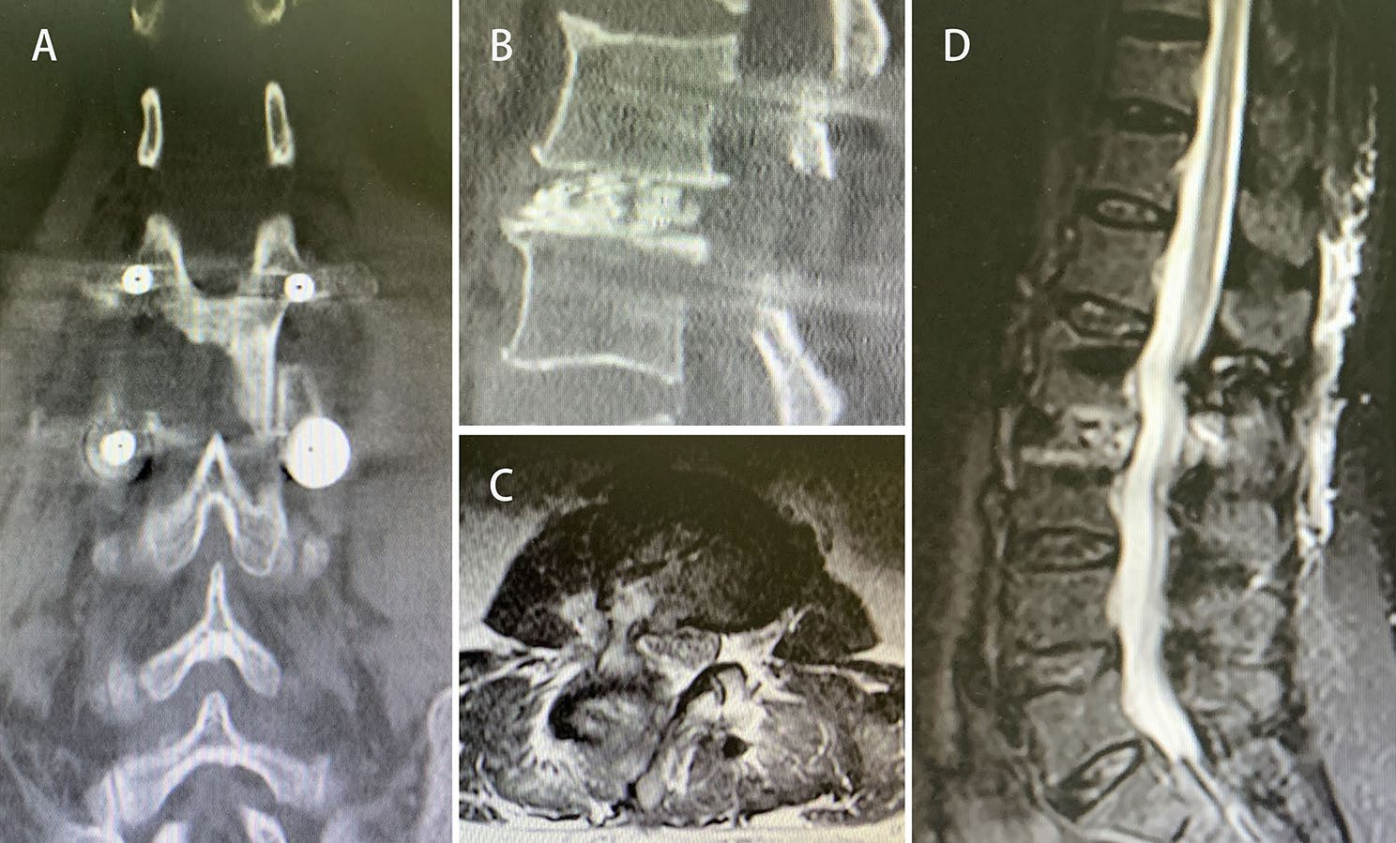
Postsurgical CT and MRI showing sufficient intervertebral bone grafting and complete decompression. (**A**) Extent of laminectomy in coronal CT. (**B**) Sufficient intervertebral bone grafting in sagittal CT. (**C** and **D**) Complete decompression in MRI images

The postsurgical imaging data, depending on the patient’s recovery, were obtained 1–3 days after the surgery. The herniated nucleus pulposus was completely removed using either the interlaminar approach or the paraspinal approach to restore the spinal canal volume. For non-fusion cases, the scope of bone tissue resection was small, and no obvious iatrogenic instability was noted after the surgery (Figs. 3, 4, 5, 6, 7, 8, 9, 10 and 11).

### Scores of VAS and ODI

The VAS scores of patients with low back pain and leg pain significantly improved at different time points after the surgery compared with those before the surgery, and the difference was statistically significant. The ODI score was similar to VAS score, and the difference in scores before and after the surgery was statistically significant (Table 2).

**Table 2:** Comparison of VAS and ODI at each time point

Time	VAS in back pain	VAS in leg pain	ODI (%)
Presurgical	5.75 ± 1.29	6.82 ± 1.93	63.84 ± 12.46
Postsurgical 1 week	2.86 ± 1.01	3.11 ± 1.37	36.51 ± 9.50
Postsurgical 1 month	2.04 ± 1.10	2.00 ± 1.12	27.36 ± 10.58
Postsurgical 3 months	1.68 ± 0.98	1.54 ± 1.20	18.26 ± 7.80
Final follow-up	0.82 ± 0.72	0.86 ± 0.97	7.26 ± 4.48
*F*	154.67	158.26	232.23
*P*	0.000	0.000	0.000

### Clinical efficacy

All patients got out of bed 1–3 days after the surgery, and the postsurgical hospital stay was 8.28 ± 4.22 days. The postsurgical follow-up time was 15.82 ± 4.54 months. Moreover, 24 patients with non-fusion surgery returned to work within 3 weeks after the surgery, and 4 patients who underwent single-space interbody fusion surgery returned to work or daily life within 3 months after the surgery. At the last follow-up, the ratio of excellent to good rate was 96.43% according to the modified MacNab score, including excellent in 24 cases, good in 3 cases, and fair in 1 case.

### Complications and treatment

Two patients (in non-fusion cases with interlaminar approach) experienced postsurgical numbness and pain in the lower limbs without muscle strength and reflex abnormalities. They recovered within 1 month after treatment with low-dose methylprednisolone combined with a pregabalin capsule and methycobal.

## Discussion

### Clinical efficacy and advantages of UBE technology in treating ULDH

UBE technology belongs to the category of percutaneous spinal endoscopy. Compared with the traditional technology of posterior lumbar interbody fusion or laminectomy, the entrance of the work and view portals of the UBE technology crosses the intermuscular space to avoid stripping the muscle attached to the spinous process, damaging the posterior branch of the spinal nerve, and leading to denervated atrophy of the local multifidus. It reduces the incidence of chronic low back pain after the surgery. The UBE technique obtains a clear surgical field by magnifying the field of view, maintaining proper water pressure blood pressure control, and applying a plasma radiofrequency wand for hemostasis [13]. This study showed that the average amount of bleeding during the removal of the nucleus pulposus for ULDH was 510 mL with open posterior approach surgery and 190 mL with microendoscopic discectomy [14]. However, accurate estimation of the amount of intraoperative bleeding in UBE surgery is difficult because it flows out together with irrigation fluid. In this study, the UBE technology was considered to be more minimally invasive and associated with less bleeding according to the postsurgical drainage volume. Maintaining proper water pressure during the surgery not only inhibited the bleeding at the surgery interface but also effectively reduced the expansion of the dural sac so that a space was formed between the dural sac and the ligamentum flavum, thus reducing the risk of spinal dura injury when the ligamentum flavum was removed [15]. In this study, no complication of dural sac tear occurred. In addition, continuous irrigation with normal saline removed inflammatory factors secreted locally by surgical stimulation, reducing the occurrence of local inflammatory response and alleviating pain among patients [16]. Compared with single-portal endoscopy, the UBE technology used two portals, including the view portal and the work portal. An independent portal was used for surgical surgery, which effectively broadened the surgical field of vision and made the surgery more flexible. The direction of the portal could be adjusted according to needs, and the scope of surgery was expanded to avoid visual field blindness caused by the obstruction of a single portal [17–19]. For cases of ULDH with central canal stenosis, even contralateral nerve root compression, the UBE technique could be used to remove the protruding nucleus pulposus, while the posterior hyperplastic ligamentum flavum and part of the base of the spinous process could be removed to expand the central canal as needed. The contralateral traversing nerve root and exiting nerve root could be easily observed and decompressed using a 30° lens to truly achieve the decompression of the “three nerve roots” and achieve satisfactory clinical results.

### Key points and experience in treating ULDH with UBE surgery using two different approaches

The key points were as follows. (1) Before fluoroscopy, the intervertebral space was made perpendicular to the ground by adjusting the operating table. The entrances of the two portals were identified and marked. (2) The patient’s blood pressure was controlled at 90–110 mm Hg/50–70 mm Hg and maintained according to the patient’s basic blood pressure. (3) The irrigation solution used was isotonic saline with the plane 70–100 cm higher than the operating plane or 30–50 mm Hg of the water pressure, which kept flowing smoothly. (4) In the interlaminar approach, the initial target point of the endoscope and the instrument was located at the junction of the spinous process and vertebral lamina of the superior vertebral body, while the initial target was located in the isthmus for the paraspinal approach. (5) In the paraspinal approach, adjusting the maximum visual field of the 30-degree endoscope to the direction of the spinal canal to discover and resect of the paracentral or centrally prominent nucleus pulposus tissue. (6) Before the surgery was completed, the irrigation solution was discontinued and the surgical field was observed for 1 min. In the case of any bleeding, the solution was completely stopped. (7) The lavage fluid was squeezed and drained from the surgical area when the surgery was complete. A drainage tube was left in the portal site.

Two patients in this group experienced postsurgical numbness and pain in the lower limbs without muscle strength and reflex abnormalities caused by excessive traction of the nerve root during the surgery; however, they disappeared 1 month after conservative treatment. Compared with the lower vertebral body, the lamina inclination angle of the upper lumbar vertebra was larger and the lamina space was smaller. Therefore, the range of laminectomy using the interlaminar approach was more extensive when the UBE technique was employed. The paraspinal approach was used according to the surgical indications, with a smaller osseous resection range and a lower incidence of iatrogenic instability. In this study, the paraspinal approach was used in six cases with satisfactory results, and no obvious instability was found in the postsurgical imaging examination. For the paraspinal approach, the isthmus was selected as the first visual field. The exit roots tended to be located anterior to the surgical field of view and lacked the coverage of the ligamentum flavum in some cases. Care was taken not to damage the anterior exit root when removing the local bone. In addition, the nerve root artery was usually located in the axilla of the exit root, and pre-hemostasis was performed in this area to avoid massive hemorrhage caused by the surgery, resulting in blurred vision and delay of the surgical process.

### Study limitations

The current study is a retrospective study. Its purpose is to introduce a minimally invasive surgical technique without setting up a control group. This study lacks a large sample and multi-center study, and the follow-up time is short.

## Conclusion

The application of UBE technology in ULDH surgery showed the advantages of clear vision and sufficient decompression. Central or paracentral intervertebral disc herniation can be treated with an interlaminar approach. For patients with foraminal or extraforaminal disc herniations, a paraspinal approach may be used to achieve good clinical results. The short-term follow-up results showed that the curative effect was satisfactory.

## Data Availability

The datasets used and/or analyzed during the current study are available from the corresponding author on reasonable request.

## Abbreviations

UBE: Unilateral biportal endoscopy
ULDH: Upper lumbar disc herniation
ODI: Oswestry disability index
VAS: visual analogue scale
SAP: Superior articular process
